# Catastrophic Antiphospholipid Syndrome

**DOI:** 10.1155/2016/4161439

**Published:** 2016-06-07

**Authors:** Rawhya R. El-Shereef, Zein El-Abedin, Rashad Abdel Aziz, Ibrahim Talat, Mohammed Saleh, Hanna Abdel-Samia, Amro Sameh, Mahmoud Sharha

**Affiliations:** ^1^Rheumatology Department, Faculty of Medicine, Minia, Egypt; ^2^Neurology Department, Faculty of Medicine, Minia, Egypt; ^3^Cardiology Department, Faculty of Medicine, Minia, Egypt; ^4^ICU Department, Faculty of Medicine, Minia, Egypt; ^5^Radiology Department, Faculty of Medicine, Minia, Egypt

## Abstract

This paper reports one case of successfully treated patients suffering from a rare entity, the catastrophic antiphospholipid syndrome (CAPS). Management of this patient is discussed in detail.

## 1. Introduction

The antiphospholipid syndrome (APS) is a systemic autoimmune disease characterized by the occurrence of arterial and venous thrombosis and/or obstetric complications (miscarriage, fetal death in utero) associated with the presence of antiphospholipid antibodies [1]. The catastrophic antiphospholipid syndrome (CAPS) is the most severe manifestation; CAPS is rare affecting less than 1% of patients with APS and is characterized by the occurrence of thrombosis in multiple organs over a short period of time [2]. It is acute in onset, with majority of cases developing thrombocytopenia, less frequently hemolytic anemia, and disseminated intravascular coagulation. Lupus anticoagulant and anticardiolipin antibodies have been reported as predominant antibodies associated with CAPS. The mortality rate is approaching 50%. It was first recognized in patients with systemic lupus erythematosus (SLE) and later found in association with other autoimmune disorders. This condition has also been recognized as a syndrome that can develop independent of any underlying disease, known as primary APS [3]. The high rate of mortality should warrant greater awareness among clinicians for timely diagnosis and treatment of this life-threatening condition. We report a case of a 32-year-old female admitted with clinical and laboratory findings consistent with CAPS which were successfully treated. Their management was discussed in detail later.

## 2. Clinical Case

A 32-year-old Egyptian physiotherapist female patient was admitted in the ICU of Al-Rashid Hospital, complaining from altered mental status, dyspnea, fever, and epileptic fit. The patient had no medical problems and had been in her usual state of health until 3 days before admission and then she developed frequent resistant headaches and blurring vision; CT of brain was done and was normal before admission in ICU by 3 days. This patient was admitted under neurology and received phenytoin and antibiotics vials. She was diagnosed as status epilepticus. After 2 days the patient experienced worsening of her neurological condition, presenting drowsiness, diplopia, squint, reduction of vision, severe headache, and developed quadriparesis. The patient was deteriorated into spastic quadriplegia within few hours. CT of brain showed slightly ill-defined hypodense lesions in both parietal lobes with faint blood density in left sided and white matter edema are noted. MRI of brain showed bilateral venous sinus thrombosis. The patient was subjected to the following laboratory: HIV antibody, HIBs antigen, which was negative. Blood culture showed no growth. D-dimer test was negative. INR was 0.88%. Platelet count was low, 100.000 mm^3^. All liver, renal function, and electrolyte were normal. An initial diagnosis of bilateral stroke was retained. The treatment started by IV drip of heparin in maximum dose with no response clinically; also INR was not affected. The dose of oral anticoagulant (warfarin) was increased up to 11 mg with IV heparin with INR ranging from 1.2 to 1.3% in normal range.

After complete history taking from her husband, we found that there was past history of fetal miscarriage and history of abortion and intrauterine fetal death. There was history of preeclampsia in first baby with blood pressure of 200/110 during her delivery. In all her pregnancies, she received juspirin and low molecular weight heparin (clexane vial). There was also past history of receiving oral contraceptive pill for one year before this attack. There was family history of death of two of her relatives from preeclampsia during labor. Then the patient referred to consultant of rheumatology to complete the management.

Complete neurological and rheumatological examinations were done; there was spastic quadriplegia. There was severe abdominal tenderness with tenderness in right hip and right lower limb. The patient's vision was deteriorated rapidly, with developing diplopia and severe headache.

At that time, brain imaging demonstrated an increase in the feature discussed before. Echocardiography showed signs of cardiac failure with aortic, mitral, and tricuspid valve insufficiencies but no segmental hypokinesia. Laboratory tests showed moderate inflammation (CRP at 30 mg/L); D-dimer test was negative. Lupus anticoagulant (LA) and anticardiolipin IGG and IGM were done in higher centre, and positive PTT-LA was 162.3 seconds (reference value: 32–51 seconds in this laboratory), DRVV time (dilute Russell's viper venom time) was 99.5 seconds, and screen ratio was 2.51 (reference value not more than 1.5 in this laboratory). Thrombin time was more than 240 seconds (reference value: 14–22 seconds in this laboratory) and anti-beta 2GP1 IgG antibodies were positive and measured 33 AU/mL (Arbitrary Units/mL, reference < 20 AU/mL). Elevated LDH was 650 U/L (reference value: 225–400 U/L in our laboratory). Anticardiolipin IgG and IgM were negative. Antinuclear antibodies and the anti-dsDNA antibodies were negative, with normal complement and normal P and C ANCA.

The diagnosis of CAPS was suspected, and our therapeutic strategy combined curative anticoagulation with unfractionated heparin, and methylprednisolone (sulomedrol vial) was given as pulse dose at dose of 1 g/d for 5 days. Then prednisone was maintained at 1 mg/kg/day with daily intravenous immunoglobulin of 400 mg/kg/5 days with physiotherapy 2 times daily. With this treatment, evolution was rapidly favorable. Neurological symptoms regressed within few days, spasticity was absent, the movement of limbs started more in left side, and the muscle strength increased gradually, as well as dyspnea. Inflammatory parameters and thrombocytopenia were corrected within few days.

After 10 days from starting of medication of sulomedrol and gamma globulin the patient developed severe pain and swelling in right lower limb. Doppler for arteries and veins of right lower limb was done and showed totally thrombosed external iliac and common femoral, deep femoral, and popliteal vein with no colour flow inside, with mild subcutaneous oedema at the leg and calf region. Anterior and posterior tibial veins and peroneal vein were patent. Doppler ultrasound of all arteries was normal.

The patient received full anticoagulation again with the maximum time of 10 days and heparin IV drip 35000 IU daily with addition of dabigatran (pradaxa tablet 110 mg twice daily). Treatment with methylprednisolone was repeated by the same regimen, and this was relayed by a maintenance dose of 2 mg/kg/day. Cyclophosphamide 1 g was also added to the initial regimen. The administration of cyclophosphamide was repeated monthly for six months. With this treatment, the initial evolution was favorable. The right lower limb for first time started to move and strength of muscle reached grade 3 in few days.

After 15 days from beginning of second regimen of medication, Doppler of both lower limbs was done again and showed evidence of recanalization of the previously described deep venous thrombus of the right lower limb with sluggish blood flow inside. Left common femoral vein and deep femoral vein were patent with normal blood flow inside. But there was thrombosed left popliteal vein with no blood flow inside. Arterial supply was normal in both lower limbs.

Recurrence of thrombosis made us take the decision of doing filter to avoid pulmonary embolism. So, we stopped heparin infusion; the patient was maintained on pradaxa (thrombin inhibitor) and warfarin tablet 13 mg daily, and then the patient was shifted to do inferior venae cava filter in Al-Kaser El-Aini Hospital, Cairo university.

The first elevation in INR was after 10 weeks from starting steroid and cyclophosphamide. INR reached 2.8 then 3 then 3.5 percent. We started to decrease the dose of warfarin gradually after 3 months from initial treatment especially after autoantibody started to decrease in her blood.

So, during her stay in the ICU (45 days), the patient presented multiple thrombotic complications: bilateral venous sinus thrombosis, thrombosis of the right external iliac, common femoral, deep femoral, popliteal vein, and left popliteal vein. Also, there was avascular necrosis in right hip which was diagnosed by CT scan. There was diplopia with blurring of vision which was suggestive of retinal thrombosis.

Patient currently has improved significantly since her initial presentation. Her vision has improved allowing her to see shades of light and shapes and she has not had any further thrombotic episodes on clinical presentation and imaging. She is now able to ambulate while she was spastic and quadriplegic at presentation (after 3 months, she walked on crutches and then walked with cane and then walked without support after 6 months). Her LDH has normalized to 166 U/L. Of particular note is that improvement in patient's symptoms, visual acuity, and functional status as well as LDH has occurred after receiving medication. Lupus anticoagulant started to decrease after 2 months and the INR started for the first time to increase. The first symptom of this patient started on 11 April 2015; on October 2015, she went to her work as physiotherapist without any support. Now, on 1 May 2016, the patient stopped steroid and finished cyclophosphamide; she received warfarin sodium 8 mg only without any thrombosis in any organ.

## 3. Discussion

Antiphospholipid syndrome is a systemic autoimmune disorder characterized by arterial and/or venous thrombosis and recurrent fetal loss and can be associated with thrombocytopenia [3]. CAPS, a fatal variant of APS, was first described in 1992 and defined as thrombosis of at least three different organ systems over a very short period of time with histopathologic evidence of multiple small vessel occlusions and high titers of antiphospholipid antibodies (APL) [3–7].

The clinical manifestation of CAPS depends on the organ involvement affected by thrombosis. The major organs involved during the catastrophic episode were renal (71%), followed closely by lung (64%), brain (62%), heart (51%), and skin (50%) [8]. Our patient presented with epilepsy, cerebrovascular accident, spastic quadriplegia, blurring of vision with diplopia, acute avascular necrosis in right hip with acute DVT in both lower limbs, heart involvement, thrombocytopenia, and severe hypertension. All these organs were affected within few days. Interestingly, our patient's hypertension was initially worked up as a possible etiologic source for our patient's severe disease manifestation. However, it has been reported and also implemented in the preliminary criteria for classification of CAPS that hypertension tends to occur in conjunction with renal involvement [7].

Laboratory findings in CAPS patients may include thrombocytopenia, hemolytic anemia which is often accompanied by schistocytes, and disseminated intravascular coagulations (DIC) [8]. The autoantibodies of interest to diagnose APS are anti-B2-glycoprotein which was detected by enzyme-linked immunosorbent assay (ELISA), anticardiolipin (aCL), or lupus anticoagulant (LA) assay [3, 9]. The recent 2006 revised classification criteria for APS updated the timeframe for the presence of elevated titers of antiphospholipid antibodies from >6 weeks to its persistence for >12 weeks [1, 9]. In our study, lupus anticoagulant was highly positive during the admission and six weeks after being hospitalized and then transient decrease of antibody started. Review of the literature was unrevealing regarding length of time it may take to develop recurrent positive antibody testing after treatment, given that our patient has lost the antibody (LA delta 6.3–7.7 seconds) transiently after 12 weeks. It may also be worth researching this timeline further as repeating antibody testing prior to 12 weeks may be sufficient.

Laboratory studies have become important diagnostic criteria for detecting APS. LA activity is detected by coagulation assays that adhere to guidelines from the International Society of Thrombosis and Haemostasis (ISTH), updated in 2009 by Pengo et al., which include (a) prolonged phospholipid-dependent coagulation time found on a screening test (activated partial thromboplastin time and dilute Russell's viper venom time), (b) failure to correct prolonged coagulation time during mixing studies, (c) correction of prolonged coagulation time found on screening test by adding excess phospholipids, and finally (d) exclusion of other coagulopathies [3, 10]. The screening test criteria cutoff in the updated ISTH guidelines includes cutoff value above 99th percentile of the distribution [10].

Diagnosis and aggressive therapies are mandatory. Besides treating the trigger, including infection, specific treatment targets thrombosis and SIRS (systemic inflammatory response syndrome). Early anticoagulation is of utmost importance regardless of the severity of thrombocytopenia [11]. Intravenous unfractionated heparin is usually preferred to low molecular weight heparin in case of DVT. If the patient has a lupus anticoagulant, monitoring is based on heparin blood level instead of aPTT. Heparin is followed by VKA for an INR of approximately 3. Corticosteroids inhibit aPL-mediated thrombosis [12]. Some authors suggest very high doses (1 g/day of methylprednisolone) before a relay with usual doses of 1 mg/kg·day methylprednisolone equivalent. Duration depends on the clinical response. Intravenous immunoglobulin (IVIG) is proposed instead of plasma exchanges in case of hemodynamic instability [13, 14], usually at a dose of 2 g/kg in 4 to 5 days [6, 9]. The combination of anticoagulation with corticosteroids and IVIG provides 69% of success [15]. Some treatments have a specific indication. In our case cyclophosphamide was used successfully in treatment of CAPS monthly. Cyclophosphamide is indicated only in refractory CAPS, particularly in the presence of lupus flare [16].

## 4. Conclusion

Catastrophic antiphospholipid syndrome is a rare disease with high mortality. Its early identification is crucial in order to establish an effective treatment: anticoagulation, corticosteroids, and immunoglobulin. Cyclophosphamide could be a promising treatment in cases of refractory CAPS. We have used it successfully for our patient. Careful postpartum anticoagulation management is mandatory. Dabigatran, a new oral direct inhibitor of thrombin, has prevented thrombus development through direct, competitive inhibition of thrombin (thrombin enables fibrinogen conversion to fibrin during the coagulation cascade). It also inhibits free and clot bound thrombin and thrombin-induced platelet aggregation. Currently well-defined indications for treatment of DVT and pulmonary embolism in patients who have been treated with a parental anticoagulant for 5–10 days and to reduce the risk of recurrence of DVT and PE in patient who has been previously treated and its prescription outside these specific contexts should be argued. The dabigatran may be a new line of anticoagulation in treatment of DVT in catastrophic antiphospholipid syndrome. Also, cyclophosphamide gives successful result in treatment of CAPS. Contraceptive pill is serious risk factor for thrombosis in APS patient.
